# Reconstructing the immune system with lentiviral vectors

**DOI:** 10.1007/s11262-017-1495-2

**Published:** 2017-07-25

**Authors:** Henning Olbrich, Constanze Slabik, Renata Stripecke

**Affiliations:** 1 German Center for Infection Research, Partner Site Hannover, Hannover, Germany; 2 Regenerative Immune Therapies Applied, REBIRTH Cluster of Excellence Hannover Medical School, Hannover, Germany; 3 Department of Hematology, Hemostasis, Oncology and Stem Cell Transplantation, Hannover Medical School, OE6862, Hans Borst Zentrum, Carl Neuberg Strasse 1, 30625 Hannover, Germany

**Keywords:** Lentiviral vectors, Hematopoietic stem cells, T cells, TCR, CAR-T cells, Dendritic, Cells, Cancer, Chronic infections

## Abstract

Lentiviral vectors (LVs) developed in the past two decades for research and pre-clinical purposes have entered clinical trials with remarkable safety and efficacy performances. Development and clinical testing of LVs for improvement of human immunity showed major advantages in comparison to other viral vector systems. Robust and persisted transduction efficiency of blood cells with LVs, resulted into a broad range of target cells for immune therapeutic approaches: from hematopoietic stem cells and precursor cells for correction of immune deficiencies, up to effector lymphoid and myeloid cells. T cells engineered for expression of chimeric antigen receptors (CARs) or epitope-specific transgenic T cell receptors (TCRs) are in several cancer immune therapy clinical trials worldwide. Development of engineered dendritic cells is primed for clinical trials for cancer and chronic infections. Technological adaptations for ex vivo cell manipulations are here discussed and presented based on properties and uses of the target cell. For future development of off-shelf immune therapies, direct in vivo administration of lentiviral vectors is warranted and intended. Approaches for lentiviral in vivo targeting to maximize immune therapeutic success are discussed.

## Introduction

### Basic aspects: cell targets and mode of infection

The most outstanding feature of *Retroviridae* is their ability to integrate DNA into the host cell genome.This property can be utilized to establish expression of a delivered coding sequence persistently and stably over months with only a single transduction. Named after the genus of the original virus, there are gammaretroviral (RVs) and lentiviral (LVs) vectors. Notably, gammaretroviruses can only infect dividing cells, whereas lentiviruses integrate into non-proliferating cells as well. Among the gammaretroviruses, the human specific species mostly exist as proviruses within the genome and infections are transmitted congenitally. Exogenous infection with gammaretroviruses is rare in humans and leads to mutagenesis due to random insertion of the viral genome potentially into proto-oncogenes. Lentiviruses like the human, simian or feline immunodeficiency virus (HIV, SIV, or FIV, respectively), however, are instead usually contracted exogenously within the adult population and primarily infect cells of the immune system. Integration of lentiviruses in long-term clinical follow-up of HIV patients under combined anti-retroviral therapy (cART) was shown to be associated with clonal expansion [1]. Yet, HIV infections *per se* rarely lead to occurrence of oncogenesis. Malignancies in HIV patients are mostly a consequence of a debilitated immune system and anti-tumor immune surveillance. Ironically, LVs derived from HIV have continuously progressed in the past twenty years as a forefront platform for gene therapy for immune reconstruction [2, 3].

### Major breakthroughs for lentiviral vector development: from the proof-of-concept towards clinical production

**Fig. 1:**
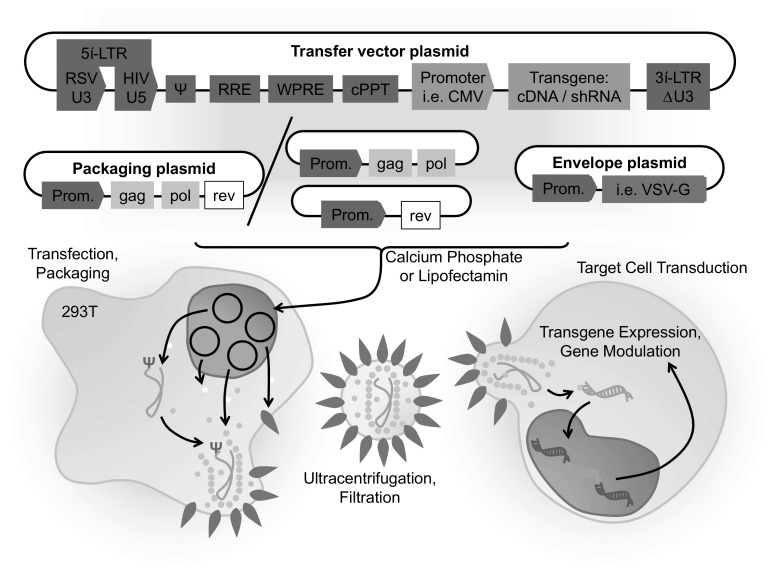
Schematic representation of the packaging plasmids and procedures for generation plasmids of self-inactivating lentiviral vector for transduction of target cells

In 1996, for the first time, HIV-based vectors were produced by splitting the viral genome among different plasmids for expression of packaging and envelope proteins and transfer of the backbone vector, which were used for transient transfection of packaging cells [4]. To broaden the target cell spectrum, VSV-G-protein is commonly used instead of HIV-envelope proteins. Unlike previously established RVs, the vectors were able to transduce terminally differentiated cells, from hematopoietic cells to neurons, broadening the range of applications for gene therapy dramatically. Later, the so-called self-inactivating (SIN) design with a 400-nucleotide deletion in the U3 region of the 3′ long terminal repeat (LTR) and including the TATA box transcriptional sequence was developed [5]. This deletion abolished the LTR promoter activity without affecting virus titer, yet improving the biosafety of HIV-derived vectors by reducing the likelihood that replication-competent retroviruses could originate in the vector producer and target cells, and hampering putative recombination with wild-type HIV in an infected host. This SIN design was remarkable, as it improved the potential performance of the vector by removing LTR sequences previously associated with transcriptional interference and also allowed the design of internal tissue-specific or regulatable promoters, which resulted into more-stringent vectors (Fig. 1). For production of high-grade clinical vectors, LV production has been in more recent years carried out under GMP conditions effectively, involving purification of the virus by ultracentrifugation and size-exclusion chromatography [6].

## Correction of immune defects in hematopoietic cells

### Lentiviral vectors showed an excellent safety profile: the case of WASP and X-SCID

**Fig. 2:**
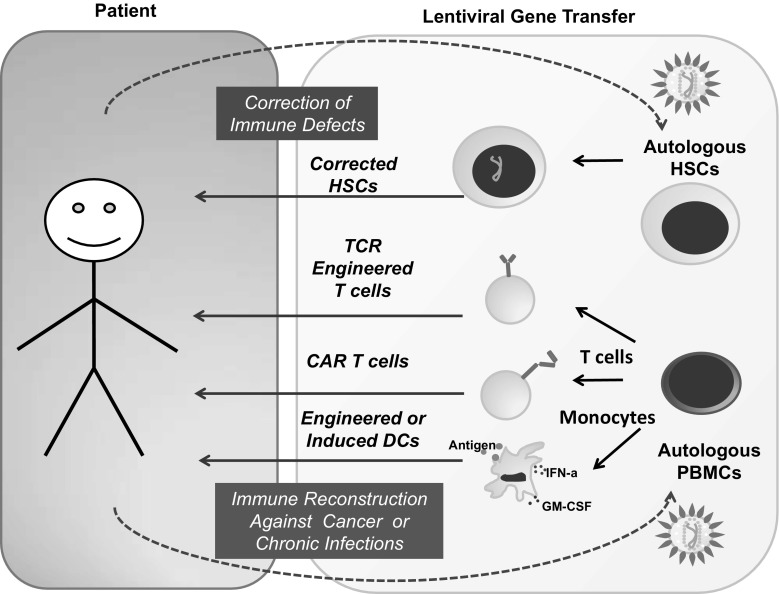
Use of patient derived hematopoietic stem cells, T cells and monocytes for ex vivo gene modification with lentiviral vectors and re-administration for gene correction and immune therapy purposes

The current standard-of-care to cure immunodeficiencies caused by germline mutations is the allogeneic hematopoietic stem cell transplantation (allo-HSCT). As donations from fully matched siblings are often not available, the alternatives are allo-HSCTs with stem cell obtained from related haploidentical or unrelated HLA-matched or HLA-mismatched donors, but those are associated with increased morbidity and mortality, e.g., causing graft versus host disease (GVHD). For that reason, great interest has been aroused in the field of genetic correction of the patient’s autologous stem cells for curing immune deficiencies (Fig. 2) (Table 1). Wiskott-Aldrich syndrome (WAS), for example, is caused by mutations in the gene encoding the cytoskeleton protein WASP. Patients suffer from eczema, highly increased susceptibility of infections, and micro-thrombocytopenia causing bleedings. Different gene therapy approaches have been studied in clinical trials all based on ex vivo genetic correction of hematopoietic stem cells using several vector systems. Transduction of peripheral blood hematopoietic stem cells with replication incompetent RV carrying the WAS protein encoding gene driven by a RV long terminal repeat (LTR) leads to multi-lineage expression following infusion in all 10 patients included in the first trial [7]. Gene therapy restored the lymphocyte functions and reduced of the incidence of autoimmunity, bleeding diathesis, and occurrence of infections. Unexpectedly, 1 out of 10 developed acute myeloid leukemia (AML) and 6 out of 10 patients treated developed T cell acute lymphoblastic leukemia (T-ALL), out of which two subsequently also developed AML. This outcome lead to severe concerns regarding RV safety and raised the need of a safer vector system. Introduction of a LV with a SIN LTR and a reconstituted WAS gene promoter was shown to decrease the risk of insertional mutagenesis and mimic physiological expression. Two clinical studies reported significant reduction of infection susceptibility and improvement overall clinical scores [8, 9]. Notably, no serious adverse events such as clonal expansions of genetically altered cells with integration sites near proto-oncogenes were observed so far in these patients.

**Table 1:** Use of LVs for corrections of immune deficiency and immune therapies: cell targets, purpose, characteristic of the target cells, and examples of therapies in development

Cell target	Purpose	Characteristic of target cell	Therapies in development	References
HSCs	Genetic correction	Quiescent	WASP	[2–5]
		Multipotent	X-LINKED SCID	[6, 7]
		Long persistence (years to life-long)	Artemis SCID	[8]
		Genotoxicity risks		
T cells	Targeting of immune responses to antigens	Naïve or memory T cell	TCRs	
		Highly replicative	Melanoma (CG)	[9, 10]
		Terminally differentiated	CAR T	
		Central memory T cells have long-term persistence (months to years)	Leukemia (CD19, CD123, FcμR, CD5)	[11–14]
		Risks of cytokine release syndrome, off-target effects	Adenocarcinoma (Tn-MUC1)	[15]
			Viral infections (gB)	[16]
Dendritic cells	Enhancement of antigenic processing and activation of adaptive responses	DC precursors (monocytes)	Cancer	
		Immature DCs	Melanoma	[17–19]
		Quiescent	Leukemia	[20]
		Short half-life (days to weeks)	Prostate cancer	[21]
		Co-expression of cytokines and maturation factors possible	Colon cancer	[22]
		Migration to lymph nodes	Chronic infections	
		Systemic administration (i.v) possible	HIV	[23]
			HCV	[24]
			HCMV	[25]
			HPV	[26]

X-linked SCID is another immunodeficiency currently being actively pursued by gene therapy. X-linked SCID is caused by mutations in the interleukin-2 receptor γ-chain gene (IL2RG). The common γ chain (γc) protein plays an important functional role in many cytokine receptors like IL-2, IL-4, IL-7, IL-9, IL-15, and IL-21 and mutations in γc can lead to defective T- and NK-cell differentiation. Patients suffer of severe susceptibility to infections as they lack functional T- or NK-cells and only poorly functioning B cells. In the past two decades, several clinical studies have been performed applying RV, SIN-RV, and LV as vectors to transfer the correct IL2RG encoding sequence to autologous HSC. Early RV vector designs used to correct γc by gene therapy resulted into insertional mutagenesis and occurrence of T-ALL in several patients [10, 11]. As these severe safety issues using retrovirus vector systems were discovered, improvements in vector safety were accomplished by introduction of SIN vector systems (SIN-RV, SIN-LV), and placement of therapeutic gene encoding sequences under the control of internal promoters like a short form of the elongation factor 1-α (EF1-α) promoter [12]. So far, no serious adverse events have been reported in these studies and reduced clustering of vector integration events around proto-oncogenes like LMO2 was observed.

In order to characterize the insertional mutagenesis and predict vector safety, the genomic integration patterns of RV and LV were studied and compared. In a study comparing SIN-LV versus RV in gene therapy for Wiskott-Aldrich syndrome, lentivirus was found to integrate into genes involved in different biological mechanisms. In contrast, RV was found to insert preferentially into genes related to the hematopoietic system. Clusters of retroviral insertion sites were found close to the MECOM and LMO2 loci. Both play important roles in transcriptional regulation and hematopoietic development and are known to be proto-oncogenes. In contrast, after SIN-LV gene transfer, insertion in these sites was found to be rare. SIN-LV integration sites were common within loci such as KDM2A, PACS1, TNRC6C, which are not known to act as proto-oncogenes and no cases of insertional mutagenesis were observed so far. In addition, histone modifications close to insertion sites of RV and SIN-LV were compared. The H3K4me3 histone modification found at transcription start sites and the H2AZ histone variant marking enhancer regions were spotted close to RV insertion sites but not around LV integration sites. Altogether, the occurrence of SIN-LV integrating into coding sequences of a broad range of different genes in contrast to RV preferentially targeting transcriptional start sites and enhancer sequences of proto-oncogenes indicated that SIN-LV could be a safer vector system [8]. Nonetheless, a close comparison between SIN-RV and SIN-LV is necessary as it was shown previously that a SIN design of RV could also improve vector safety [12].

### Corrections of additional immune defects: artemis-SCID and ADA-SCID

Artemis-SCID is a primary immunodeficiency caused by a defect in Artemis endonuclease responsible for homologous end-joining of double-strand DNA breaks. These genomic changes are caused by external factors or recombinations that occur in developing lymphocytes during the early stages of T and B cell maturation. This immunodeficiency shows a T^−^/B^−^/NK^+^ SCID phenotype. The construction of a SIN-LV encoding for the functional Artemis gene driven by a minimal human DCLRE1c 5^′^ promoter (APro) showed correction in Artemis-deficient human fibroblasts and comparable IgM and IgG production in Art^−/−^ mice transplanted with transduced HSC and WT HSC. No enrichment of integration sites at oncogenic sites was observed [13].

Another exemplar primary immunodeficiency is the adenosine deaminase SCID (ADA-SCID), which is characterized by the lack of ADA enzyme and accumulation of the deoxyadenosine triphosphate substrate. This results in impaired lymphocyte development, viability and function causing severe infections in affected patients. In studies using RVs, 31 out of 42 included patients showed clear benefit of gene therapy and recombinant ADA enzyme replacement therapy could be stopped [14, 15]. Still, immune recovery was not complete, with low T cell numbers and several patients depending further on immunoglobulin therapy. Although viral vector insertions near oncogenes have been reported, no severe safety issues such as RV-mediated insertional mutagenesis and leukemia were observed [16]. Incidentally, the European committee for human medicinal products has recently suggested marketing authorization for RV gene therapy for ADA-SCID patients when a matched related HSCT is not possible [17]. SIN-LVs encoding a wild type codon-optimized ADA gene downstream of a short form of the EF-1α promoter in were effective to transduce HSCs, which were shown to rescue ADA^(−/−)^ mice from their lethal phenotype in vivo and in in vitro assays to correct human ADA-deficient CD34^+^ cells. LV-ADA showed significantly less transformation potential compared to analogous RVs, and vector integration-site analysis of LV-transduced human cells grown in immune-deficient mice showed no evidence of clonal bias [14]. Thus, it will be interesting to compare the efficacy and long-term safety of LVs and RVs side-by-side in gene therapy clinical trials of ADA-SCID patients.

## Creation of enhanced re-targeted T cells for serial killing

### T cells expressing designer T cell receptors

Adoptive transfer of T cells form a donor matched through the human leukocyte antigens (HLAs) to a recipient has shown convincingly clinical evidence that T cell receptors (TCRs) recognize cells presenting the cognate antigenic epitopes resulting in cytotoxic T lymphocyte (CTL) function. The concept of redirecting the patients’ own cells through genetic engineering to tackle defined antigenic targets emerged during the last decade as a new era of T cell therapy (Fig. 2). This has successfully been used clinically to produce robust immune responses against various malignancies as novel treatment options. Careful consideration is however necessary in the choice of target antigens, as they should be exclusively or preferentially present on the target tumor or virus-infected cells to prevent adverse effects against normal tissues or cells.

Transduction of T cells with genes for specific engineered TCRs circumvented the much laborious methodology of expanding and characterizing the high-affinity T cells primed with antigens. T cells engineered with RVs for expression of high-affinity TCRs against cancer germline antigens have shown responses in patients with melanoma and synovial sarcoma. Here, different antigenic epitopes that are expressed in tumors and presented through the HLA are selected as targets. Clinical studies exploring RVs for expression of TCRs demonstrated severe consequences of bystander off-tumor toxicities [18, 19], largely due to mispairing of the engineered and endogenous TCRs. Thus, in an elegant ‘single TCR editing’ approach, a SIN-LV encoding an HLA-A2-restricted NY-ESO TCR transgene was combined with deletion of the TCR provided by an adenoviral vector expressing a zinc-finger nuclease. TCR-edited T cells mediated tumor rejection in a xenograft mouse model without inducing xenogeneic graft versus host disease, thus resulting in improved safety [20].

### T cells expressing chimeric antigen receptors

Chimeric antigen receptors (CARs) correspond to single-chain antibodies (serving as a recognition domain for antigens present on the surface of target cells) fused to different types of co-stimulatory domains (such as for example CD28 or 4-1BB), which in turn are fused to the CD3ζ-chain (the T cell activation domain). T cells expressing CARs (CAR-Ts) can bind directly to cell-surface antigens, thus recognizing the cell targets and killing them. Opposed to T cell expressing engineered TCRs, T-CARs bypass the need of HLA-restricted antigen presentation by target cells, which is commonly donwregulated in tumor cells. Nevertheless, CARs provide potent T cell activation and co-stimulatory signals leading to expansion, serial killing of targeted cells, and recruitment of bystander immune cells [21].

CAR-Ts targeted against CD19 (CART19) were utilized for treatment of advanced B-cell acute and chronic lymphocytic leukemia (B-ALL and B-CLL). Although expressed in these malignancies, CD19 is also present in normal B cells. Using LVs for transduction, highly efficient CAR-T cell ex vivo engineering was achieved, which was correlated with long persistence and dramatic expansion of CAR-Ts in vivo, in treated patients [22]. LV-CART19 administration led to complete remission in multiple cases as opposed to poor survival rates of patients treated under conventional regimes. Nevertheless, occasional leukemia relapse with occurrence CD19^−^ blasts escaping CAR recognition was observed [23]. Notably, relapse could be prevented by treatment of the patients with CAR-Ts dually transduced with an additional LV expressing a CD123-CAR [24]. Other explored leukemia targets under evaluation with RV and LV engineered CAR-T cells include the FcμR that is more specific to B-CLL blasts [25], and the T cell marker CD5 in T cell ALL [26]. Unlike their proven clinical potency against hematologic malignancies, CAR T cells have shown modest efficacy against solid organ tumors [27]. The reason is that solid tumors are less accessible by T cells and develop a very immune suppressive environment, and are thus this is currently a major challenge in the field. CAR T cells are also in development against chronic viral infections, e.g., human cytomegalovirus (HCMV) [28]. Nevertheless, many viruses have immunosuppressive mechanisms that can also potentially impair the function of CAR T cells.

### LVs versus RVs for T cell engineering

Introducing genes for engineered TCR or CAR expression to T cells has proven feasible with both retro- and lentiviral vectors. T cells can be driven to proliferate in vitro, but according to their in vivo physiology, some subsets of T cells have a lower potential of expansion, and are therefore more likely to be efficiently transduced with LVs than with RVs, i.e., effector memory T cells are prone to exhaustion, but establishment of memory engineered or CAR T cells is crucial for treatment of chronic diseases. In fact, CART19 produced with a LV have been shown to form a persistent memory population in patients, and that seems to contribute to prolonged control of the leukemia [22].

Because re-targeted memory T cells can potentially persist for a lifetime, safety considerations regarding the risk of insertional mutagenesis or vector replication have been considered. Fortunately, no cases of aberrant expansion of transduced T cells due to insertional activation of cellular proto-oncogenes in vivo have been reported using either LVs or RVs. Overall, re-targeting of T cells provides extremely flexible and individualized treatment strategies that have been effective against otherwise hardly treatable conditions. GMP production of RVs is currently much cheaper since stable packaging cell lines are broadly available, whereas GMP production of LVs is mostly performed by transient transfection, which is a practical factor, particularly for larger trials. Yet, it remains to be compared side-by-side in clinical trials if they show similar efficacies.

## Dendritic cells engineered for higher viability and potency to bypass immune suppression

### Delivery of antigens into DCs

Dendritic cells (DCs) are professional antigen (Ag)-presenting cells (APCs) expressing high levels of class I and class II HLAs. Upon antigen uptake and activation, DC can initiate Ag-specific immune responses to prime naïve T helper cells and CTLs and also boost memory T cells. With considerable advances related to the method of tumor Ag loading into DCs and maturation stimuli, they have become promising tools for cancer immunotherapy, which opened a broad development of clinical protocols. After numerous smaller clinical trials since 1990, large phase III trial are currently ongoing for immunotherapy of melanoma, prostate cancer, and glioma exploring Ag-loaded DC vaccines as sole or as adjuvant cell therapy [29].

Since the early 2000s, transduction of human or mouse DC and their precursors with LVs showed consistently high and persistent transgene transfer capabilities (reviewed in [30]). Thus, the concept that monocytes or DCs could be obtained from patients, genetically modified and re-administered, has continuously evolved (Fig. 2). Notably, unlike lytic-type viral vectors such as adenovirus, LV-gene transfer showed no reduced DC viability or expression of unwanted viral antigens. Besides, retroviral vectors were not shown effective to transduce terminally differentiated, non-replicating primary monocytes, and DCs. Since then, LVs have been intensively explored for immunization approaches, either as vectors to reprogram DC vaccines with antigens and potentiating immune modulatory molecules or as a novel viral vaccine modality *per se* (reviewed in [3, 31]). LVs expressing a large variety of different types of antigens such as, for example, viral proteins (such as human immune deficiency virus Gag Pol and Rev [31], hepatitis C virus structural and non-structural protein [32], human cytomegalovirus pp65 [33]) or tumor associated antigens (such as melanoma TRP2 or MART-1 [30], leukemia WT1 [34], colon cancer MUC-1 [35]) were validated. Several studies by many groups including ours explored in vitro T cell stimulation assays [32, 34, 36], syngeneic mouse models [30, 37], mice with adoptive human T cells [34, 38], and also mice transplanted with a fully humanized immune system [39]. These studies showed that, provided that these antigens were not toxic to the 293T packaging cells or to the dendritic cells, LVs expressing full-length antigenic proteins up to 7 kilobases could be efficiently expressed and processed in DCs for presentation by class I and class II MHC. Therefore, unlike loading DCs exogenously with peptide epitopes, the efficacious internal loading with LVs offered a substantial advantage, because several MHC/HLA types could be loaded simultaneously and persistently. This consistently resulted into a multipotent class I and II mediated activation of CD8^+^ and CD4^+^ T cells, respectively, independent of the HLA-type of the patient. Thus, LV-mediated gene delivery into professional APCs developed into a broad personalized immunization strategy to overcome the vastly debilitated immune competence encountered in patients with cancer and with chronic infections.

### Manipulation of co-stimulatory ligands and check-points in DCs transduced with LVs

Another issue in cancer immune therapy is the immunosuppressive environment observed particularly in advanced metastatic disease. Here, lowered co-stimulation or check-point signals impinged by the tumor play a fundamental role, as they function as an “immunologic break,” inhibiting DC/T cell interactions. The DC co-stimulatory ligands CD80 and CD86, for example, interact with the cognate CD28 molecule or with the CTLA-4 receptor to activate or dampen T cell activation, respectively. During tumor development and chronic T cell stimulation, there is a shift in T cells to express CTLA-4, which results into a T cell blockade, or, so-called check-point. 4-1BB is a co-stimulatory molecule transiently up-regulated by all CD8^+^ T cells following activation, whereas 4-1BB ligand (4-1BBL) is highly expressed on activated DCs. 4-1BB provides a higher expansion of human cytolytic CD8^+^ T cells than CD28. LV expression of 4-1BBL combined with influenza nucleoprotein (NP) in mouse DCs showed induced maturation of bystander, non-transduced cells, both in vitro and in vivo, in draining lymph nodes [40]. Therefore, inclusion of 4-1BB in the LV expressing an antigen is a potential strategy to enhance immune effects *in trans*. Similarly, CD40 ligand (CD40L) is a co-stimulatory molecule expressed by activated T helper cells important for DC maturation upon engagement with its receptor CD40. When human DCs were transduced with LV for ectopic expression of CD40L, a potent maturation resultant from a autocrine feed-back regulation was observed, resulting in much higher levels of antigen-specific T cell activation in vitro [41].

### Lentiviral-induced dendritic cells

Although dendritic cell vaccines loaded with antigens were superior in comparison to administration of antigens alone in several animal models, production of DCs were hampered in the clinical translation due to its complex, personalized and lengthy manufacturing. Monocytes are the preferred source of DC precursors, quite abundant in the peripheral blood (15–25%), but they have a short half-life of a few days. When monocytes are incubated with combinations of cytokines such as GM-CSF/IL-4 or GM-CSF/IFN-α, they acquire typical DC features (high MHCII, CD80, CD86 expression) and functions (high potential to activate and expand memory and naïve T cells). However, upon cytokine deprivation, ex vivo grown-DCs rapidly lose their viability. This is one of the main issues found in the several clinical trials performed worldwide with DCs, which showed only modest therapeutic effects against cancer (such as melanoma, leukemia) and chronic infections (HIV, HCMV). As an alternative to ex vivo culture with recombinant proteins, the LV-mediated introduction of cytokine genes into monocytes resulted into their self-differentiation into highly viable and potent “induced DCs” (iDCs) (for a review see [42]). Several proof-of-concept animal models demonstrated the potency and safety of LV-iDCs for immune therapy of melanoma [37, 43, 44], leukemia [34], and development of adaptive T and B cell responses against the HCMV pp65 antigen in immune-deficient mice transplanted with human hematopoietic stem cells [33, 39]. Upscaling the production of integration competent and integrase defective LVs (IDLVs) and generation DCs was readily achieved under good-manufacturing practices (GMP) [33, 44, 45]. Notably, although pre-clinical data showed substantial promise, the induced DC field is considerably less developed regarding their clinical use than genetic correction of HSCs and T cell enhancement with TCRs and CARs. One of the main hypothetical concerns for clinical development of engineered DCs is the risk that these cells would result into uncontrollable autoimmunity or insertional mutagenesis and myeloid malignancies. However, long-term evaluation of iDCs in syngeneic and humanized mouse models did not show these adverse effects (Sundarasetty et al. in preparation). In addition, integration of LV and IDLV in the genome of iDCs showed a benign polyclonal pattern, with no involvement of the insertional mutagenesis hot-spots observed in malignancies caused by retroviral vectors [44, 45]. However, a practical concern for the clinical development of iDCs is still the high cost of production and testing of LVs under GMP. Therefore, other simpler gene-transfer modalities, mostly based on RNA transfer into DCs, are the currently most sought technologies for clinical development. RNA-mediated expression of antigens and cytokines is however transient, lasting hours to a few days, and this short antigenic effect still remains to be confirmed for clinical efficacy in larger randomized clinical trials.

## Direct local or systemic administration of LVs as a novel modality for viral vaccines or gene therapy

### Bio-distribution of VSV-G pseudotyped LVs, in vivo gene transfer and proof-of-concepts

The use of RVs applied at high concentrations systemically or intra-tumorally as a medicament was actively explored in the early 2000s [46, 47]. These studies were eventually discontinued due to the low efficacy of in vivo RV gene transfer, possibly due to the fact that the vectors used were of sub-optimal concentration or few non-replicating cells could be effectively transduced (or both). Contemporarily, direct administration of LVs in vivo as a novel viral vaccine entity or “cell-free” gene therapy started to take place. Initial studies demonstrated that systemic intravenous administration of LVs pseudotyped with the VSV-G envelope were notably efficacious to infect MHC class II^+^ cells (mostly DCs and B cells) of spleen, bone marrow, and liver of immune-competent mice [48]. Subsequently, LV vaccines progressed in different types animal models demonstrating proof-of-concepts upon expression of tumor antigens for immunotherapy against cancer, such as melanoma [48] and prostate cancer [49], or expression of viral proteins as vaccines against chronic viruses such as HIV [50] and human papilloma virus (HPV) [51]. For direct administration aimed to correct genetic defects in hematopoietic cells, VSV-G pseudotyped LVs expressing wt ADA showed bio-distribution in bone marrow, spleen, liver, and lung, and satisfactory ADA expression efficacy in mice and monkeys for potential clinical development [52]. Although these exciting studies showed so far no apparent malignancies in animal models, the regulatory agencies in the United States and in Europe demand that if vector sequences are integrated in the host, then clinical protocols with the product require clinical long-term follow-up observations.

### Engineering new viral envelopes to target-specific cells

A rational approach to reduce the risk of insertional mutagenesis and malignancy could be to avoid the gene transfer into hematopoietic stem cells or progenitor cells (since these are long lived cell and more prone to oncogenesis) and restrict the gene delivery specifically to mature, terminally differentiated type cells. Therefore, use of LV direct administration by different routes to genetically correct or improve cells would be, at least hypothetically, safer if the cells in question would be terminally differentiated. The VSV-G protein used for pseudotyping the viral LV envelope has a broad tropism, mediated through cell-surface heparan sulfate which is found from stem cells to terminally differentiated cells [53]. Alternatively, LVs can be engineered for targeting different cell-surface markers or receptors for cell type-specific entry. Targeting ligands displayed on the vector particle surface (such as a peptide, single-chain antibody, or engineered virus proteins) capable of binding to the target surface protein can this way mediate cell type-specific transgene transfer. Although this exciting field has been pursued for several years, it was only recently that re-targeted LVs could be consistently produced without compromising the LV infectivity titers and validated in direct in vivo gene delivery [54]. Buchholz et al. have described LVs targeted to T cells expressing tumor Ag-specific TCR or CARs using engineered glycoproteins of measles virus (MV) incorporated into the envelope membrane of LV particles. Cell-type specificity is provided through a single-chain antibody (scFv) that recognizes the CD8^+^ or CD4^+^ cell-surface antigen [55, 56]. Other types of approaches are currently be explored for targeting of dendritic cells for immunotherapy purposes. Use of heavy-chain-only antibodies found in llamas that were immunized human DCs was described (“nanobody display technology”) [57]. Nanobodies were screened by consecutive rounds of cellular panning and flow cytometry characterization on human DCs and were efficiently incorporated in the packaging of LVs.

## Conclusions

In this review, we aimed to provide a broad showcase for the use of LVs to reconstruct the immune system: from correcting genetic defects in HSCs to engineered powerful effector T cells and, further, more potent genetic vaccination strategies. The vector technologies that have evolved in the past twenty years were incrementally advanced towards optimizing packaging and the gene cargo, restricting expression to certain target cell types and enhancing safety. Unfortunately, these cutting-edge LV technologies are taking a long time to be implemented into clinical trials. Notably, safety aspects have been intensively scrutinized: analyses of LV integration sites in the genome showed a benign profile, replication-competent lentivirus (RCLs) were not detectable, and possible unwanted malignancies were not observed in animal models or in clinical trials. Incidentally, animal models have shown high efficacy and potency for therapeutic use, with an excellent safety profile. As these issues have been quite well addressed, a current major impeding practicality is the high cost for production of LVs for clinical trials, since only a few academic centers and companies are equipped to generate and test clinical-grade LVs. How this impediment can be overcome will depend on new biotechnical progress and competitiveness. Besides, larger clinical trials currently in progress or in preparation will ultimately foment a large industrial interest for the biomedical use of LVs towards immune reconstructive goals.
